# ‘”Why me, why now?” Using clinical immunology and epidemiology to explain who gets nontuberculous mycobacterial infection

**DOI:** 10.1186/s12916-016-0606-6

**Published:** 2016-03-23

**Authors:** M. Alexandra Lake, Lyn R. Ambrose, Marc C. I. Lipman, David M. Lowe

**Affiliations:** Royal Free London NHS Foundation Trust, London, UK; Division of Infection and Immunity, University College London, London, UK; Institute of Immunity and Transplantation, University College London, Royal Free Campus, Pond Street, London, NW3 2QG UK; UCL Respiratory, Division of Medicine, Faculty of Medical Sciences, University College London, Royal Free Campus, London, UK

**Keywords:** Nontuberculous mycobacteria, Host defence, Primary immune deficiency, Interferon gamma, Interleukin 12, Bronchiectasis, Cystic fibrosis, Immune response

## Abstract

**Background:**

The prevalence of nontuberculous mycobacterial (NTM) disease is rising. An understanding of known risk factors for disease sheds light on the immunological and physical barriers to infection, and how and why they may be overcome. This review focuses on human NTM infection, supported by experimental and *in vitro* data of relevance to the practising clinician who seeks to understand why their patient has NTM infection and how to further investigate.

**Discussion:**

First, the underlying immune response to NTM disease is examined. Important insights regarding NTM disease susceptibility come from nature's own knockouts, the primary immune deficiency disorders. We summarise the current knowledge surrounding interferon-gamma (IFNγ)-interleukin-12 (IL-12) axis abnormalities, followed by a review of phagocytic defects, T cell lymphopenia and rarer genetic conditions known to predispose to NTM disease. We discuss how these define key immune pathways involved in the host response to NTM. Iatrogenic immunosuppression is also important, and we evaluate the impact of novel biological therapies, as well as bone marrow transplant and chemotherapy for solid organ malignancy, on the epidemiology and presentation of NTM disease, and discuss the host defence dynamics thus revealed. NTM infection and disease in the context of other chronic illnesses including HIV and malnutrition is reviewed. The role of physical barriers to infection is explored. We describe how their compromise through different mechanisms including cystic fibrosis, bronchiectasis and smoking-related lung disease can result in pulmonary NTM colonisation or infection. We also summarise further associations with host factors including body habitus and age.

**Summary:**

We use the presented data to develop an over-arching model that describes human host defences against NTM infection, where they may fail, and how this framework can be applied to investigation in routine clinical practice.

## Background

Nontuberculous mycobacteria have historically been seen as environmental organisms of limited clinical relevance, overshadowed by their more aggressive cousin, *Mycobacterium tuberculosis.* It was not until the HIV pandemic highlighted disseminated *Mycobacterium avium* and *intracellulare* as major opportunistic infection syndromes that their significance was recognised by the general healthcare community, a role further cemented by the expansion of iatrogenic immunosuppression. Evidence to support an ongoing rise in disseminated NTM infection is limited [1]. This is not the case for chronic pulmonary NTM disease which is increasing [2] in part due to an aging, vulnerable population [3, 4].

NTM are regarded generally as low pathogenicity organisms, which can be transiently isolated from samples such as sputum, colonise body sites such as the lung, or cause persistent infection and disease. Distinguishing between these different clinical states can be surprisingly difficult. However, it is important to do as this underpins both clinical management decisions and predicts outcome. An accepted approach is to define NTM-associated pulmonary disease as that in which compatible clinical features occur in people from whom NTM are repeatedly isolated over time [5]. Treatment can be poorly tolerated and is certainly less effective than that for tuberculosis (TB) [6]. Hence an understanding of who is at risk enables us to both target interventions and potentially prevent disease occurring.

Research into monogenic disorders conferring susceptibility to disseminated NTM infection provides key insights into the critical host immune responses against these organisms, though many questions remain: in particular why such a large population of apparently immunocompetent people become infected and develop pulmonary disease.

We present here a summary of how specific conditions and iatrogenic interventions have elucidated both the essential and redundant components of human defences against NTM. We use this to suggest practical strategies for investigation in patients presenting with these infections.

## Discussion

### Lessons from primary immune deficiencies

CLINICAL VIGNETTE*: A 2-year old boy born to first-cousin parents presents with several weeks of fever, weight loss, diarrhoea and skin nodules. Examination reveals widespread lymphadenopathy and hepatosplenomegaly. Blood cultures are positive for M. avium.*

A number of monogenic disorders conferring susceptibility to disseminated NTM infection are grouped together as Mendelian Susceptibility to Mycobacterial Disease (MSMD) conditions. Although extremely rare [7] and predominantly affecting children, these diseases offer insight into critical immune defences against mycobacteria.

#### Cytokine pathway defects

**Fig. 1:**
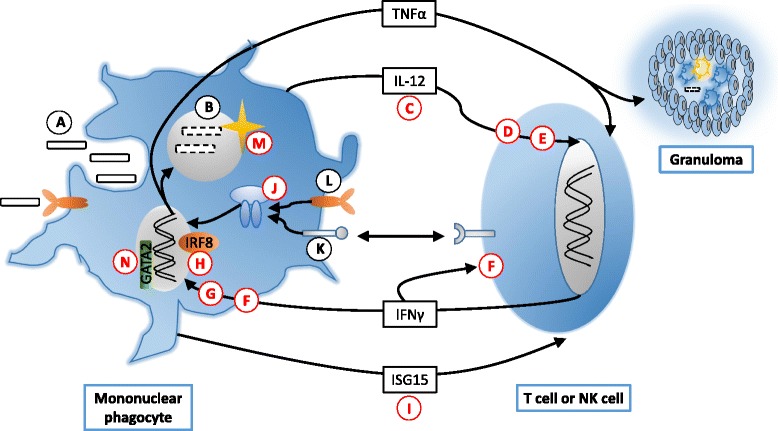
The immune response to mycobacterial infection and known sites of dysfunction. Human genetic syndromes which affect the immune response to mycobacterial infection are known to result from disorders in the following genes: ISG15, IL-12B, IL12RB1, IFNGR1, IFNGR2, STAT1, IRF8, ISG-15, GATA2 and NADPH oxidase complex subunit genes such as CYBB. Nontuberculous mycobacteria (A) are phagocytosed (B), triggering release of IL-12 (C), a heterodimeric cytokine formed from the gene products of IL12A and IL12B, which binds a receptor heterodimer (D) of IL-12RB1 and IL-12RB2 on T cells and NK cells. Signalling to the nucleus mediated by TYK2 (E) then results in IFNγ production. IFN gamma binds its receptor (F), a heterodimer of IFNGR1 and IFNGR2, triggering phosphorylation of JAK2, JAK1, and STAT1 (G). The resultant phosphorylated STAT1 molecule homodimerises to form the pSTAT1 complex which translocates to the nucleus and binds the IFN gamma activating sequence. This triggers transcription of interferon stimulated genes (ISG) via IRF8 (H), and increases IL12, TNFα, ISG15 (I), and potentiation of macrophage activation. Activated macrophages demonstrate enhanced phagosome maturation and increased killing of intracellular pathogens, and upregulated antigen presentation, thereby activating Th1-phenotype T cells to proliferate and release further IFNγ. TNFα drives development of granulomas. IRF8 aids differentiation of myeloid progenitors into monocytes, and controls transcriptional responses of mature myeloid cells to interferons (IFNs) and Toll-like receptor (TLR) agonists. NFκB is a rapid-acting transcription factor modulated by NEMO (J) and activated by stimuli including signalling through CD40 (K), TLR (L), reactive oxygen species and TNFα. Activation allows a host of inflammatory and immune responses, including IL12 release. Effective intraphagosomal killing through reactive oxygen species requires an intact NADPH oxidase complex (M). Intact haematopoiesis of monocyte lineages is also required via GATA2 (N)

The immune response to mycobacterial infection is summarised in Box 1 and Figure 1, together with sites of dysfunction due to primary immunodeficiency syndromes. Essentially, defence is mediated by mononuclear phagocytes’ ability to kill mycobacteria and secrete IL-12, augmented by IFNγ-secreting lymphocytes (especially CD4^+^ T cells).

Increased susceptibility to mycobacterial disease is seen in several human genetic syndromes which result from mutations in critical cytokine pathways affecting IL-12β [8, 9], IL-12Rβ1 [10], IFNγR1 [11–13] and IFNγR2 [14]. Mutations in the transcription factor STAT1 [15–19] result in failure to respond to signals from type I (IFNα/β), type II (IFNγ) or type III (IFNλ1/2/3) interferons, and bring a dual risk of mycobacterial and viral infections, as can deficiency of tyrosine kinase 2 (TYK2), a janus kinase widely active in cytokine signalling. Loss of mycobacterial infection control in this latter condition is caused by loss of the intracellular signalling cascade triggered by IL-12 and mediated by the interaction of IL-12Rβ1 with TYK2 [20]. Mutations in interferon regulatory factor (IRF)-8 [21] impair production of IL-12 in response to IFNγ. Mutations in interferon-stimulated gene (ISG)-15 [22] also confer susceptibility to NTM: the gene product, interferon-induced 17 kDa protein, is involved in IFNγ production by T and NK cells. In some of these genetic syndromes, mycobacterial infection has been successfully treated with adjunctive interferon administration [23–26].

Newly described mutations in RAR-related Orphan Receptor C (RORC) predispose to mycobacterial infection as well as mucocutaneous candidiasis. RORC encodes the *ROR*γ and *RORγT* isoforms of a transcription factor involved in regulating immune function, cellular differentiation and metabolism. Although susceptibility to candidiasis is due to disturbances in the IL-17 pathway, the increased risk of mycobacterial infection is again via impaired IFNγ responses (in this instance from γδ T cells and CCR6^+^CXCR3^+^CD4^+^ αβ Th1 cells) [27].

Deficiency of nuclear factor‐κB essential modulator (NEMO) results in NTM susceptibility within the diverse phenotype of this X-linked condition, implicating NF-κB signalling and by extrapolation the upstream messenger TNF and/or signalling via Toll-like receptors (TLRs) [28, 29].

#### Phagocyte defects

Defence against intracellular pathogens such as NTM requires effective intracellular killing by phagocytes including neutrophils, monocytes, macrophages and dendritic cells. In chronic granulomatous disease (CGD), the respiratory burst - critical to phagocyte activation and intracellular killing - is impaired by the lack of functional NADPH oxidase.

A variable proportion of CGD patients (6-57 %, study-dependent) develop local and/or systemic complications following vaccination with bacillus Calmette-Guérin (BCG) [30]. The importance of the macrophage respiratory burst in defence against mycobacteria is highlighted by a mutation resulting in a reactive oxygen species formation defect in macrophages but not neutrophils that is nevertheless still associated with susceptibility to tuberculosis [31].

Intriguingly, although there is evidence from *ex vivo* experiments that human neutrophils restrict the growth of or kill many NTM species [32–34] and that neutrophils contribute to the control of *M. avium* in mice [35–37], there is little to suggest that patients with isolated neutrophil disorders or neutropenia have a specifically increased risk of NTM infection.

#### Other primary immunodeficiency syndromes

Autosomal dominant deficiency of the transcription factor GATA-2 carries a significant risk of NTM infection [38]. This disorder has a diverse presentation, such that it may be diagnosed at any time from early infancy to old age. Manifestations can vary from the asymptomatic to near-lethal. Typical features include monocytopenia, B and NK cell cytopenias, and myelodysplastic syndrome. Although multiple factors may contribute to NTM susceptibility, including NK cell deficiency and impaired cytokine release [39], the simplest explanation is a deficiency of mononuclear phagocytes [40, 41].

Immunodeficiencies that profoundly affect the number or function of T cells also predispose to NTM infection (as well as many other pathogens). This includes Severe Combined Immune Deficiency (SCID) [42] but also isolated CD4^+^ T cell deficiency [43], which is associated with both pulmonary and disseminated infection, emphasising the importance of this T cell type.

Some primary immunodeficiencies are not associated with a significantly increased risk, including the antibody-deficiency syndromes, such as Common Variable Immunodeficiency (CVID) [44] and X-linked Agammaglobulinaemia (XLA) [45]. The occasional NTM infections which do occur in such patients are generally associated with significant bronchiectasis - itself an independent risk factor, as described later.

### Interferon-γ autoantibodies

Another characterised immune defect affecting host defence against NTM is anti-IFNγ autoantibody formation [46, 47]. A cohort of NTM patients, frequently of Asian origin and over 60 years old, have been demonstrated to produce these antibodies [48–50]. Analysis typically demonstrates loss of IFNγ-mediated augmentation of TNFα and STAT1 phosphorylation in whole blood, which normalises when serum is removed, consistent with the presence of inhibitory antibodies against IFNγ [50]. Notably, patients with treatment-refractory disseminated NTM infection and IFNγ autoantibodies have been successfully treated with anti-CD20 (Rituximab) and experienced sustained remission from infection [51, 52].

Autoantibodies against IL-12 are also described, especially in association with thymoma [53]. These may be expected to increase risk of NTM infection, though their clinical significance remains unclear at present. Importantly, autoantibodies including those directed against cytokines, tend to be more prevalent in older adults [54].

### Immunological insights from NTM patients and genome-wide associations

Many of the disorders discussed so far represent fundamental defects in key antimycobacterial pathways, and often manifest as disseminated infection in young people. However, older patients with isolated pulmonary NTM (p-NTM) infection may have more subtle abnormalities of the same immunological processes, such as impaired IFNγ (and often TNFα) production [55–57] in stimulated whole blood or peripheral blood mononuclear cells. There are also reports of clinical improvement with the administration of IFNγ in patients with pulmonary MAC infections [24, 58].

However, these findings are not consistently replicated: in a prospective cohort study of 63 adults with p-NTM, no significant abnormality of immune function was found in the IL-12/IFNγ axis or numbers of lymphocyte subsets [59]. Studies of other cytokines in NTM disease including TGF-β, IL10, IL-17, and IL-18 reveal a differential balance relative to controls [60, 61]. This may indicate that multiple subtle immunological abnormalities co-exist in some patients that together increase overall risk. In another example of this, a recent report found an increased frequency of potentially significant polymorphisms in immune system genes (including IRF8 and STAT1) among NTM patients compared with their relatives or healthy controls [62]. One gene where polymorphisms are enhanced in NTM is NRAMP1, whose product improves resistance to intracellular pathogens by decreasing intraphagosomal iron and increasing production of inducible nitric oxide synthase (iNOS) [63]. Deleterious mutations in such a pathway would be consistent with the core immunological defence against mycobacteria: that is, a requirement for functional mononuclear phagocytes (especially macrophages) to kill mycobacteria, functional CD4^+^ T cells plus intact production of, and response to, IFNγ, IL-12 and TNFα.

Beyond intrinsic defects in host immunity, there are several other risks for NTM infection. These are summarised in the following sections.

### Systemic illness

CLINICAL VIGNETTE: *A 35-year old patient with significant graft-versus-host disease, eight weeks after unrelated-donor stem cell transplant for acute myeloid leukaemia, presents with fevers and malaise. Blood cultures from an indwelling central venous catheter are positive for M.fortuitum.*

A number of systemic illnesses increase the likelihood of NTM infection. This association can generally be understood in terms of the key immune responses describe above. For example, there is a clear and long-established relationship between disseminated NTM infection (especially *M.avium/intracellulare*) and HIV-1-seropositivity, with the risk increasing sharply when the CD4^+^ T cell count falls below 50/mm^3^ [64]. There is also an increased risk of isolated pulmonary disease, especially with *M.kansasii* [65], whose pathogenicity and clinical manifestations more closely resemble tuberculosis than other NTMs. Again, the CD4^+^ T cell count is usually low [66].

Recipients of solid organ transplants are particularly vulnerable to NTM, consistent with their broad disease-related and iatrogenic immunosuppression [67–70]. Similarly, stem cell transplant recipients, who have a profound deficiency of all lymphoid and myeloid cell types, are at significantly increased risk of NTM infection. This includes rapid-growing species which can cause central catheter infections [71]. Notably, the majority of infections probably occur outside periods of neutropenia [72], again confirming relative redundancy of neutrophils in NTM defence.

Other diseases, for example rheumatoid arthritis, are associated with heightened risk of NTM. This may relate to intrinsic T cell dysfunction [73] but also to the use of immunosuppressive medications, associated lung disease or reduced leptin levels – all discussed below. Diabetes, which is an established risk factor for TB [74] has, curiously, not been implicated in NTM infection to date.

### Iatrogenic immunosuppression

#### Targeted therapies

As described, TNFα is essential for granuloma formation. Correspondingly, there is a significant risk of developing either pulmonary or disseminated NTM infection with anti-TNFα treatment [75]. An analysis of the United States Food and Drugs Administration database of adverse events associated with TNFα inhibitors revealed over 100 confirmed and probable cases of NTM infection in that population. The most common organism was *Mycobacterium avium,* and 44 % of cases demonstrated extrapulmonary disease [76].

Other biologic and newer small molecule synthetic therapies are less well studied, but theoretical risks – based on their impact versus the key NTM defences detailed above – are discussed in Box 2 and Table 1. Those agents with high associated risk (++ or +++) are more likely to predispose to disseminated NTM disease than those with modest or low risk, although concomitant immunocompromise from underlying conditions or adjunctive treatments would compound the effect of any agent. All implicated therapies may predispose to pulmonary infection, especially in patients with structural lung disorders. Current best practice for clinicians considering prescribing biologic immunosuppressive agents is to carry out a screen for latent TB prior to initiating therapy. We suggest that consideration should also be given to detecting NTM infection (via sputum culture) prior to treatment with high-risk molecules, especially in patients with underlying pulmonary disease.

**Table 1:** Potential effects of targeted small molecule/monoclonal agents on risk of NTM Infection

		Element of NTM immune response targeted?				
Target	Example(s)	T cells	Mononuclear phagocytes	Key cytokines	Other Th1 cytokines	Theoretical risk for NTM †
TNF	Infliximab Certolizumab Adalimumab Etanercept Golimumab	N	N	Y	N	+++
IL-12/23	Ustekinumab	N	N	Y	N	+++
JAK	Ruxolitinib Tofacitinib	?	Y	Y	N	+++
CD52	Alemtuzumab	Y	Y	N	?	++
CD25/IL2R	Basiliximab Daclizumab	Y	?	N	?	++
CD3	Muromonab	Y	N	N	N	++
α4-integrin component	Natalizumab	Y	Y	N	N	++
IL-6R	Tocilizumab	N	N	N	Y	+
CTLA-4 (agonist)	Abatacept Belatacept	N	Y	?	Y	+
IL-1R1/IL-1β	Anakinra Rilonacept	N	N	N	Y	+
CD30	Brentuximab	Y	N	N	N	±
RANKL	Denosumab	Y	Y	N	N	±
CD20	Rituximab * Ofatumumab *	N	N	N	N	-
BLyS/BAFF	Belimumab *	N	?	N	N	-
Btk	Ibrutinib *	N	?	N	N	-
HER-2/erbB	Trastuzumab Pertuzumab Erlotinib	N	N	N	N	-
VEGF/VEGFR	Bevacizumab Ranibizumab Aflibercept Axitinib Lapatinib	N	N	N	N	-
EGFR	Panitumumab Afatinib Gefitinib	N	N	N	N	-
BcrAbl	Bosutinib Nilotinib	N	N	N	N	-
C5	Eculizumab	N	N	N	N	-
IgE	Omalizumab	N	N	N	N	-
GPIIb/IIIa	Abciximab	N	N	N	N	-

* Target B cells – note risk of hypogammaglobulinemia and secondary bronchiectasis

† +++ very significant risk, ++ significant risk, + some risk, ± unclear risk, − no risk

Y = Yes, N = No, ? = possible

TNF, tumour necrosis factor; IL, interleukin; JAK, Janus activated kinase; CD, cluster of differentiation; CTLA-4, cytotoxic T-lymphocyte-associated protein 4; RANKL, Receptor activator of nuclear factor kappa-B ligand; BLyS, B lymphocyte stimulator; BAFF, B cell activating factor; Btk, Bruton’s tyrosine kinase; HER-2, Human epidermal growth factor receptor 2; VEGF(R), vascular endothelial growth factor (receptor); EGFR, epidermal growth factor receptor; C5, complement component C5; IgE, Immunoglobulin E; GPIIb/IIIa, Glycoprotein IIb/IIIa; NTM, nontuberculous mycobacteria; Th1, T helper 1

#### Broadly acting immunosuppressive agents

Within the adult NTM disease patient population there is a considerable burden of chronic respiratory disease (discussed below). Inhaled and oral steroids are frequently used for treatment of many of these conditions, and a corresponding increased susceptibility to disease is found. In a Danish population-based study of respiratory disease, use of inhaled corticosteroids in COPD increased the odds ratio for p-NTM disease from 7.6 to 19.6, and a dose-risk response was noted with increasing doses [77]. A relationship with steroids and rising NTM disease risk is also seen in the treatment of rheumatoid arthritis [78] and asthma [79].

NTM disease has been associated with immunosuppressive medications such as azathioprine, cyclophosphamide, mycophenolate and cyclosporine, as well as with anti-TNFα agents [78]. Again, clinicians should consider screening for NTM before starting these drugs and remain vigilant for infection and disease during treatment.

### Structural lung disease

CLINICAL VIGNETTE: *A 76 male smoker with known COPD was investigated for new changes on his chest radiograph associated with increased sputum and a gradual reduction in his exercise capacity. He had not responded to several short courses of antibacterials. Sputum cultures isolated M. xenopi with no initial evidence for underlying lung neoplasia.*

Discussion thus far has focussed on immunological defences against NTM infection, but it is clear that the physical barriers integral to healthy lungs are also critical. In p-NTM disease case series, chronic respiratory diseases such as bronchiectasis and COPD are common associations. Equally, NTM prevalence in bronchiectasis is high [80] - estimated at 9.3 % according to a recent meta-analysis [81]. Given that lung damage, such as cavitation or bronchiectasis, is often regarded as an integral component of p-NTM disease diagnosis [5], it can be difficult to determine whether lung structural changes predispose to, or arise as a consequence of, p-NTM disease. Although non-CF bronchiectasis is a diverse entity with multiple aetiologies, a unifying patho-mechanism is provided by Cole’s vicious cycle model [82] where local pulmonary damage results in non-clearing infection that leads to an excessive inflammatory response, with consequent further lung damage (dilatation and destruction, i.e. bronchiectasis) and more infection.

The complexity of predisposition to p-NTM disease is demonstrated by a whole-exome sequencing study of 69 immunocompetent p-NTM disease patients and 18 unaffected family members. This revealed that patients with p-NTM have more low-frequency, protein-affecting variants in immune, cystic fibrosis transmembrane conductance regulator (CFTR), cilia, and connective tissue genes than their unaffected family members and control subjects [62], demonstrating the complexity of predisposition to NTM disease.

#### Chronic obstructive pulmonary disease

COPD is a chronic, progressive lung disease characterised by airflow limitation with poor reversibility. Lung injury, triggered most often by smoking, causes inflammation, tissue destruction and remodelling of elastin and collagen, with occlusion of small airways by narrowing, obliteration and mucus plugs [83], The heightened susceptibility to infection was demonstrated in a prospective cohort of COPD exacerbations where 22 % of subjects were culture-positive for NTM [84]. The extent to which NTM infection itself may promote COPD has been investigated, and might partly explain the apparent high frequency of reported mycobacterial isolation [85]. Both bronchiectasis and COPD are characterised by neutrophilic inflammation [86, 87]. As described previously, this cell type is probably not central to NTM defence, but instead can cause host damage via the release of cytotoxic contents and thereby further compromise physical barriers to infection.

#### Cystic fibrosis

Specific mutations in the CFTR gene, most commonly the delta-F508 mutation, lead to defective function of the cyclic-AMP stimulated chloride channel in the membrane of epithelial cells and the clinical disease of cystic fibrosis (CF). Homozygosity for these mutations results in disordered sodium and chloride transport across the epithelium with thickened respiratory secretions. The heightened risk of pulmonary infection is well established, and has a complex aetiology ranging from impaired mucociliary function and viscous, inspissated secretions, to compromise of many immunological defences [88].

The predisposition to NTM infection is striking: CF respiratory cultures exhibit a 10,000 fold greater prevalence of NTM than the general population, with the most common being *Mycobacterium avium* complex (MAC) and *M. abscessus* [89]. The importance of studying this population is highlighted by the reported transmissibility of *M. abscessus* from patient to patient [90]. The implication of this for people at high risk of NTM disease using healthcare services with other NTM patients is considerable. Importantly, there are an increasing number of reported CFTR polymorphisms that do not result in frank CF but nonetheless may predispose to bronchiectasis and NTM infection [62, 91–93].

Other associations with structural lung disease are summarised in Box 3. In all of these cases, it remains difficult to determine how much of the NTM colonisation, infection and disease relate to anatomical changes and secretions that are hard to clear, and how much to associated dysregulated immune and inflammatory responses.

### Other host traits as risk factors for NTM infection

Aging appears to increase susceptibility to p-NTM disease (the mean age at presentation in a US study was 68.2 years [94]). Age greater than 65 is also associated with worse outcomes (hazard ratio for death 9.17, 95 % confidence interval 4.98-16.86 [95]). Whilst this may simply reflect the fact that predisposing factors for NTM infection, such as structural lung disease, are more common with age, immunosenescence [96–99] affecting key host defences (especially T cell function) may also be important.

Many p-NTM prevalence studies reveal a greater number of female than male patients [2, 59, 100]; females with non-CF bronchiectasis seem to be at particularly high risk [80]. A possible explanation may be the lower levels of oestrogen in post-menopausal females, as experiments in ovariectomised mice show that oestrogen enhances the clearance of MAC [101], albeit human data are inconclusive [102, 103].

Low body mass index (BMI) appears to be a risk factor for NTM infection, with a protective effect seen at higher BMIs [55, 104, 105]. Lower levels of subcutaneous fat are present in p-NTM patients compared to controls [106]. It has been suggested that the adipokines leptin and adiponectin may be responsible. Leptin, a hormone expressed by white fat cells and whose levels positively correlate with body fat, regulates satiety but also has immunomodulatory effects such as driving T cell differentiation towards a Th1 IFNγ-producing phenotype, enhancing phagocyte function and increasing TNF and IL-12 secretion [55]. Leptin deficient mice (*ob/ob*) mice have delayed clearance of *Mycobacterium abscessus* lung infection compared to wild-type mice [107]. Conversely, adiponectin is a protein that has a role in fatty acid oxidation and inversely correlates with body fat; this adipokine has immunosuppressive effects on Th1 responses [104].

P-NTM disease has been reported in a patient group characterised by greater than average height, thoracic skeletal abnormalities and mitral valve prolapse [59]. The evidence that this is related to an underlying connective tissue disorder is currently limited [108]. However, abnormalities of the thoracic skeleton such as scoliosis and pectus excavatum do appear to be more common than in patients with TB or the general population [105, 109].

Severe vitamin D deficiency appears to be associated with p-NTM disease [110], although the mechanism remains less clear than for *M.tuberculosis* [111].

### Summary

NTM are increasingly isolated from respiratory secretions, and this appears to reflect a rise in true disease. Analysis of immune function and host phenotype reveals the fundamental mechanisms of successful host defence against NTM. These are an intact IFNγ-IL-12 axis, effective phagocytosis and intracellular killing, adequate monocyte haemopoiesis and circulating CD4^+^ T cell numbers, plus an undamaged pulmonary epithelium with effective clearance of secretions. Most NTM lung disease occurs in older patients. At our current level of knowledge, the largest patient population, i.e. people with isolated lung disease, appear to have minimal evidence for clear-cut, underlying immune defects. However, a small but important group will have demonstrable and relevant alterations in their ability to control NTM infection. Furthermore, subtle immune perturbations may combine with lung damage to increase the overall risk of NTM infection and disease.

**Fig. 2:**
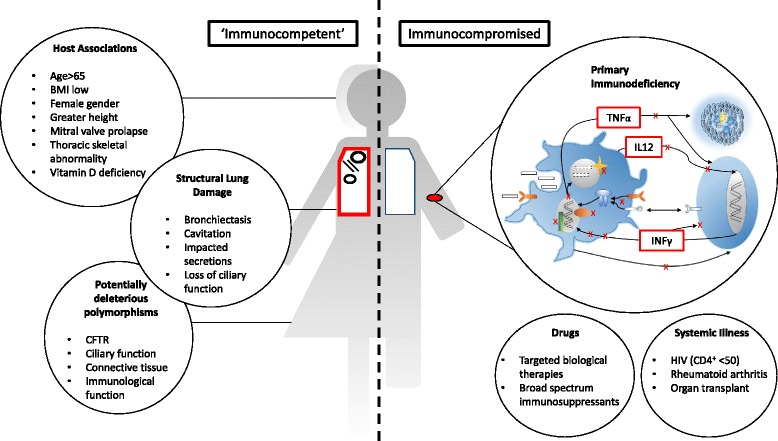
Systemic factors predisposing to NTM disease. Apparently ‘immunocompetent’ individuals may have an elevated risk of NTM disease due to structural lung disease or specific host features such as advanced age, female gender or polymorphisms of immune, cilia, connective tissue or CFTR genes. Immunocompromise causing susceptibility to NTM disease can be caused by primary immune deficiency, drugs targeting the immune system such as anti-TNFα reagents, or systemic disease

Figure 2 summarises the elements described in this article. Many of these risks are predictable and modifiable: clinicians and patients alike should strive to avoid NTM infection as its treatment is prolonged and associated with considerable morbidity [5].

**Fig. 3:**
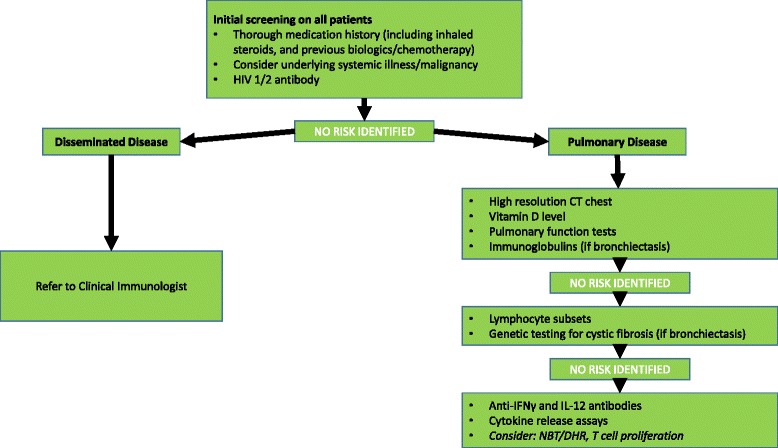
Flowchart for investigation of adult patients presenting with NTM disease

The degree to which investigations are performed to identify these risks depends on the patient’s clinical features and resources available. Figure 3 lists our advice for the investigation of adult patients presenting with NTM infection. Children or anyone presenting with apparently unexplained disseminated infection should be referred to a clinical immunologist and undergo whole exome sequencing if no defined susceptibility is identified; the pathway for investigating early-onset MSMD conditions has been described elsewhere [69].

All adult patients should be offered HIV testing and assessed for underlying disease leading to immune compromise. A careful drug history should include biologics or other therapies whose most recent administration may have been weeks or months previously. Vitamin D deficency is an easily reversible risk factor.

Clinicians should be aware in particular of the haematological characteristics of GATA-2 deficiency (monocytopenia, NK and B cell cytopenias), due to its protean manifestations and wide range of age at first presentation. Lymphocyte subsets help to identify this pattern as well as idiopathic CD4 lymphopenia.

In pulmonary infection, the wide availability of high resolution CT lung scanning at an acceptable radiation dosage means that this is now an important component of p-NTM work-up. Lung function tests will help to identify COPD in patients without a pre-existing diagnosis, and also serve as a baseline for future management. We advocate testing immunoglobulins in patients with bronchiectasis to exclude an immunological basis for the structural lung disease, even though hypogammaglobulinemia is not a particular risk for NTM infection itself. Patients with significant bronchiectasis should be investigated for underlying CF.

If no abnormalities are identified via these tests then we advocate assessing for response to, and release of, IFNγ and IL-12. Defects in these pathways can present clinically at a later age, and the tests can inform whether IFNγ may be a treatment adjunct worth considering. Anti-cytokine antibodies are more common in older patients and should be measured; if present, consideration may be given to immunomodulatory therapy (i.e. rituximab). T cell proliferation and assays for CGD should be considered if there is a compatible history, especially of other opportunistic infections.

## Box 1. Summary of the key immune responses to mycobacterial infection

Following internalisation of the mycobacterium, IL-12 is produced by the infected mononuclear phagocyte. This binds to the IL-12 receptor on T or natural killer (NK) cells, which initiates an intracellular signalling cascade culminating in IFNγ production. The secreted IFNγ then binds to its receptor on the phagocyte to signal via JAK-STAT pathways, resulting in macrophage activation and further IL-12 release, in addition to tumour necrosis factor alpha (TNFα) and interleukin 1 (IL-1). Activated macrophages demonstrate enhanced phagosome maturation, increased killing of intracellular pathogens and upregulated antigen presentation, thereby activating Th1-phenotype T cells to proliferate and release further IFNγ. The secreted TNFα plays a critical role in the development of granulomas (reviewed in [112]).

## Box 2. Risk for NTM infection from monoclonal antibodies and small molecule medications

*Anti-cytokine/cytokine signalling medications*

Of significant theoretical concern is the IL-12/23 inhibitor ustekinumab, given the central role of this cytokine in combating mycobacteria; a report has been published of TB reactivation [113], but further data are required. The JAK pathway inhibitors tofacitinib and ruxolitinib interfere with interferon signalling (as described earlier) and should also therefore carry a significant risk. Tofacitinib appears to carry a similar risk of TB to anti-TNF agents [114] and disseminated TB on ruxolitinib is described [115]. Data are currently scarce on these agents, but the immunological basis of risk for NTM infection is clear.

*B cell targeted therapies*

Although cases of NTM infection have been associated with the anti-CD20 monoclonal rituximab, reports remain rare and are usually in patients receiving other immunosuppressives [116], while the risk of TB is not significantly increased [114]. This is consistent with a relatively minor role for B cells and antibodies in host defence against mycobacteria. However, clinicians should be aware that hypogammaglobulinemia secondary to B cell-targeted therapies will increase risk of bronchiectasis and thereby colonisation or infection with NTM.

*Anti-inflammatory agents*

The anti-IL-6 agent tocilizumab and CTLA-4 agonist abatacept (which reduces co-stimulation from antigen-presenting cells to T cells) both impair Th1 responses and might be expected to modestly increase risk of mycobacterial infection. Preliminary data may support some risk of NTM, at least for tocilizumab [116, 117]; correspondingly, the incidence of TB is increased modestly with both agents but not to the level seen with TNFα blockade [114]. Molecules which interfere with the IL-1 axis (anakinra and rilonacept) similarly interrupt inflammatory responses and may modestly increase risk of NTM infection. The α4-integrin component is expressed on many leucocytes including lymphocytes and mononuclear phagocytes: the inhibitor natalizumab interferes with their migration and might increase risk of infection – as has been reported [118].

*Drugs used in malignancies and organ transplantation*

It is probably less useful to consider specific risks of agents used for malignancies and in organ transplant due to the profoundly immunosuppressive effects of the underlying conditions and adjunctive treatments. Nevertheless, molecules acting against T cell and/or mononuclear phagocyte receptors such as CD25/IL-2R (eg daclizumab, basiliximab), CD3 (muromonab) or CD52 (alemtuzumab) would all be expected to increase the risk, whereas those targeting growth factors or tissue-specific antigens would not.

*Others*

Denosumab inhibits RANKL, expressed on T helper cells and involved in differentiation of some mononuclear phagocytes, and thus may be expected to increase risk although there is no supporting data so far. This is important to establish as the population more likely to have osteoporosis (elderly women) are also at increased risk of NTM infection. In contrast, some agents target elements of the immune system not involved in defences against NTM, such as omalizumab (anti-IgE) or eculizumab (anti-complement component C5) and should not be associated with NTM infection.

## Box 3. Structural lung diseases associated with pulmonary NTM

Alpha-1 antitrypsin [119]

COPD

Cystic Fibrosis

Non-CF Bronchiectasis

Pneumoconiosis [77, 120, 121]

Pulmonary alveolar proteinosis [122]

## Abbreviations

BCG: bacillus Calmette-Guérin
CGD: chronic granulomatous disease
CFTR: cystic fibrosis transmembrane conductance regulator
CVID: Common Variable Immunodeficiency
DHR: dihydrorhodamine
HIV: human immunodeficiency virus
IFNγ: interferon gamma
IL: interleukin
MAC: *Mycobacterium avium* complex
NBT: nitroblue-tetrazolium
NEMO: nuclear factor‐κB essential modulator
NK cells: natural killer cells
NTM: nontuberculous mycobacteria
PID: primary immune deficiency
p-NTM: pulmonary nontuberculous mycobacteria
RORC: RAR-related Orphan Receptor C
SCID: Severe Combined Immune Deficiency
TB: tuberculosis
TNFα: tumour necrosis factor alpha
XLA: X-linked Agammaglobulinaemia
